# A Unicorn Disease: The Large Duct Variant of Invasive Ductal Adenocarcinoma of the Pancreas

**DOI:** 10.7759/cureus.41430

**Published:** 2023-07-05

**Authors:** Ahmed Elhariri, Jason S Starr, Sanjay Bagaria, Nguyen Tran, Hani Babiker

**Affiliations:** 1 Oncology, Mayo Clinic, Jacksonville, USA; 2 Surgery, Mayo Clinic Florida Robert D. and Patricia E. Kern Center for the Science of Health Care Delivery, Jacksonville, USA; 3 Oncology, Mayo Clinic Cancer Center, Rochester, USA

**Keywords:** hyperthermic intraperitoneal chemotherapy, cytoreductive surgery, foamy glands, large duct variant, pancreatic ductal adenocarcinoma

## Abstract

Large duct adenocarcinoma (LDA) is a rare histopathological variant of pancreatic ductal adenocarcinoma (PDAC) that closely mimics intraductal papillary mucinous neoplasm (IPMN). We present a 74-year-old female diagnosed with LDA in 2017. She was initially managed with chemotherapy and laparoscopic distal pancreatectomy. After five years of stable disease on systemic chemotherapy, she was referred to us to explore further definitive treatments. We used a multidisciplinary approach with curative-intent cytoreductive surgery and hyperthermic intraperitoneal chemotherapy (HIPEC), followed by oral maintenance chemotherapy. Subsequent scans showed stable disease; she eventually underwent neoadjuvant radiation and surgery with intraoperative radiation therapy (IORT) and achieved remission.

## Introduction

Pancreatic cancer (PC) is currently the third leading cause of cancer death in the United States with a dismal five-year relative survival of 11.5% [1]. It is expected to become the second cause of cancer death in 2030, surpassing colorectal cancer [2]. Here, we discuss a rare variant of pancreatic ductal adenocarcinoma (PDAC) referred to as large duct adenocarcinoma (LDA). It is found in only 7% of PDAC cases and defined as more than 50% of the tumor section containing infiltrative ducts with a diameter of >5 mm or macroscopically identifiable microcystic pattern; cysts are usually small with a diameter ranging from 0.5 to 0.7 cm, occasionally reaching 1 cm [3]. LDA is frequently misdiagnosed as a cystic neoplasm. Due to the rarity of this variant, there are no specific treatment guidelines to follow, so a patient-centered multidisciplinary approach was utilized in treating this patient.

## Case presentation

**Figure 1:**
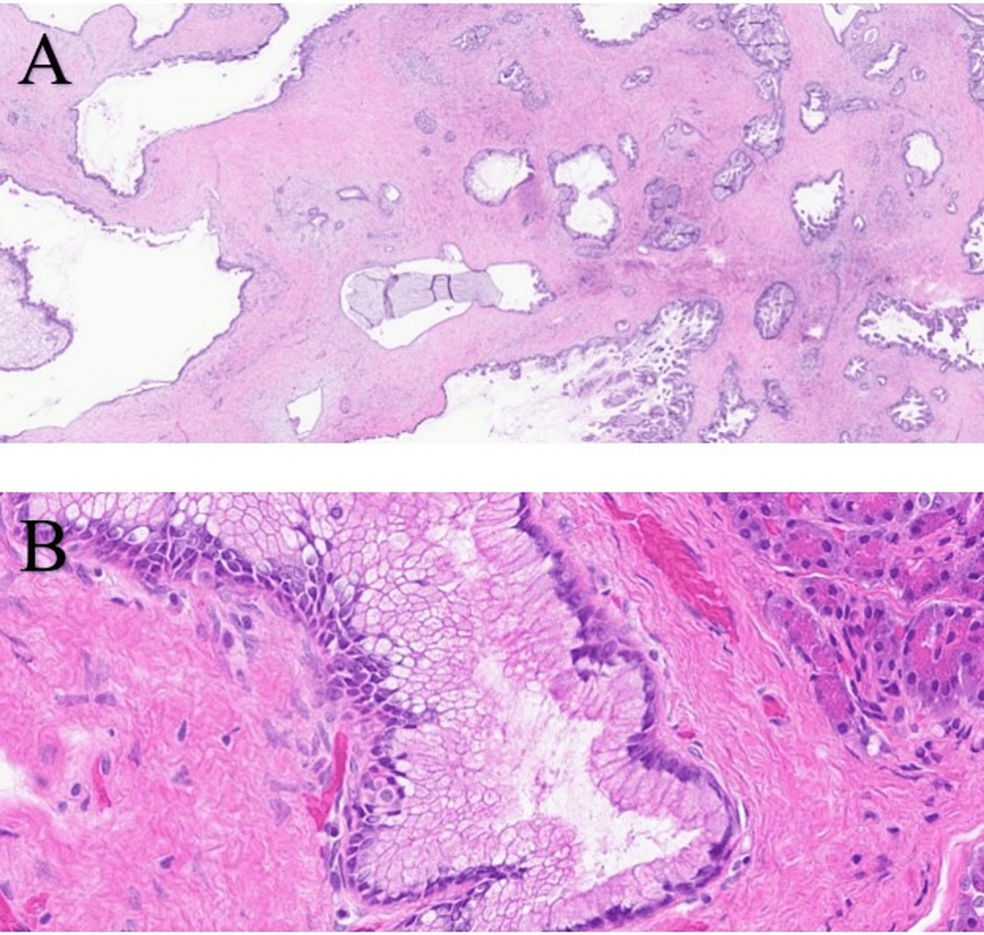
Invasive ductal adenocarcinoma of the pancreas with large duct and foamy gland patterns. A) Mature large glandular units giving the impression of native (noninvasive) ducts. However, they are numerous, having irregular and relatively disorganized distribution. They also show subtle contour irregularities and open lumen formation characteristic of LDA. B) Mucin-rich version of foamy gland adenocarcinoma showing abundant cytoplasm, distinct cytoplasmic border, brush-border-like apical condensation of the cytoplasm, and nuclear contour irregularities. Hematoxylin and eosin (H&E) staining, magnification: A) 0.8/40× and B) 40/40×. LDA: large duct adenocarcinoma

A 74-year-old female with a past medical history of gastrointestinal reflux disease and irritable bowel syndrome was referred to our cancer center. She had a family history of prostate cancer in her father and liver and urinary bladder cancers in her brother. In February 2017, the patient presented to the emergency department with nausea, diarrhea, and abdominal pain. CT imaging revealed a hypoattenuating pancreatic tail mass measuring 2.6 × 3.6 × 2.1 cm and a distal duct dilatation measuring 5 mm. This mass was biopsied using endoscopic ultrasound with fine-needle aspiration (EUS-FNA), and a few small (<3 mm) dilated side branches were observed in the pancreatic neck and body. Cytology revealed abnormal mucinous glandular epithelium suspicious of a mucinous neoplasm. Laboratory examination showed elevated carbohydrate antigen 19-9 (CA19-9, 149 U/mL; reference limit, ≤40 U/mL) and carcinoembryonic antigen (CEA, 2.9 ng/mL; reference limit, ≤2.5 ng/mL). One month later, the patient underwent laparoscopic distal pancreatectomy and splenectomy. The margins were negative, all 11 regional lymph nodes were negative for malignancy, and the spleen was tumor-free. Surgical pathology revealed a low-grade mucinous neoplasm with areas suspicious of adenocarcinoma. To reach a more definitive classification of this lesion, slides were sent for expert consultation. From the histopathological findings (Figure 1), the mass was classified as a large duct variant of invasive ductal adenocarcinoma (LDA) with a mucin-rich foamy gland pattern.

**Figure 2:**
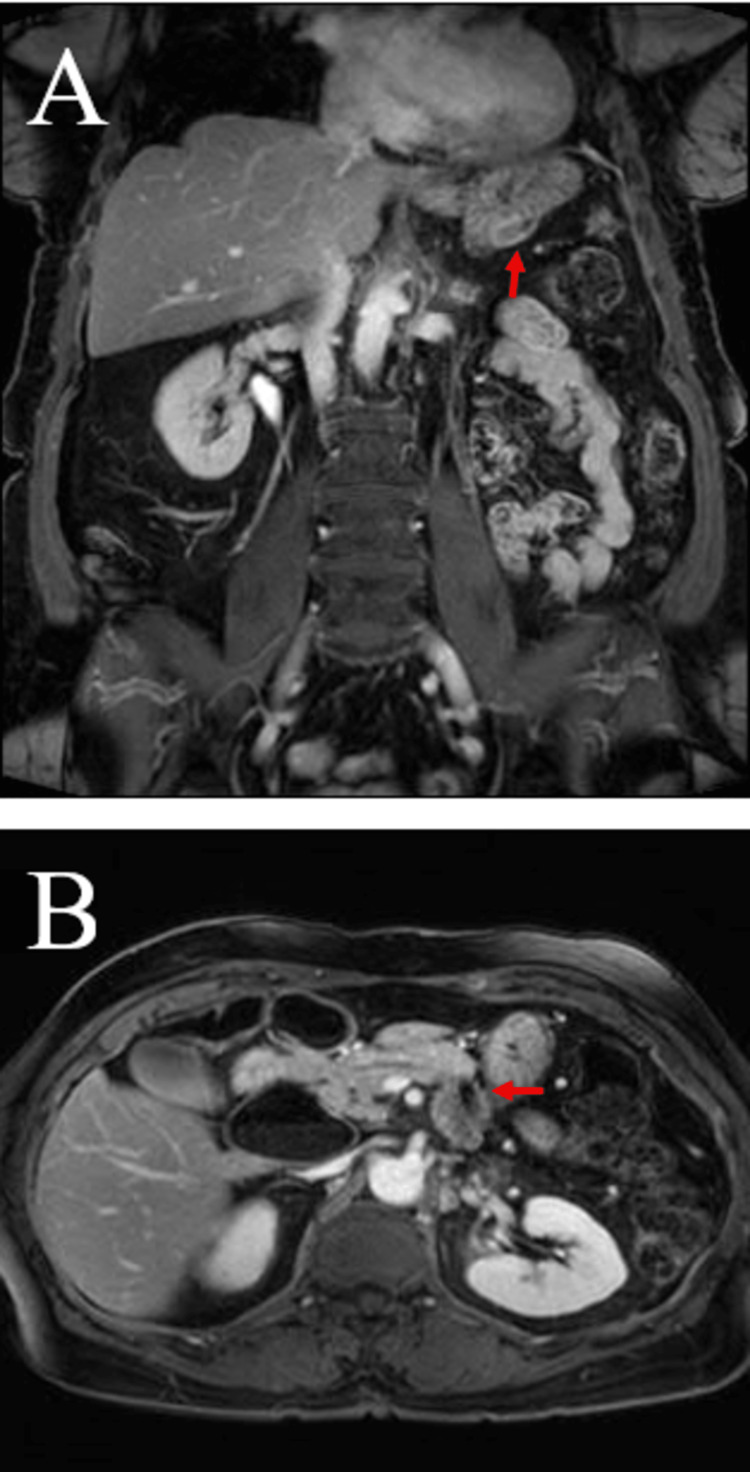
MRI of the abdomen/pelvis showing peritoneal lesions. A) 1.9 cm implant along the undersurface of the gastric body. B) 3.2 × 1.5 cm lesion along the pancreatectomy margin (previously 2.4 × 1.2 cm).

Genetic testing was performed on this specimen that showed KRAS G12R mutation, and immunohistochemical staining expressed MutL protein homolog (MLH), MutS homolog 2 (MSH2), MutS homolog 6 (MSH6), and PMS2 in the neoplastic cells (microsatellite stable). The patient started adjuvant chemotherapy with gemcitabine, and after completing six cycles (six months), she went on surveillance. Three years later, surveillance CT scan of the abdomen/pelvis detected a nonspecific mesenteric soft tissue lesion within the right lower quadrant (RLQ) and a subcentimeter peritoneal soft tissue lesion within the left upper quadrant (LUQ), both concerning for metastasis. A surgical biopsy of the LUQ peritoneal implant confirmed metastatic adenocarcinoma with mucinous features consistent with the pancreatic primary and CA19-9 (95 U/mL; reference limit: ≤40 U/mL). Subsequently, the patient started gemcitabine/protein-bound paclitaxel and had stable disease on surveillance scans. The patient was then referred to us to explore additional treatment options since she had indolent disease for five years. An MRI of the abdomen/pelvis at our center demonstrated slowly increasing metastatic implants (Figure 2), and CA19-9 was elevated (747 U/mL; reference limit: ≤40 U/mL).

**Figure 3:**
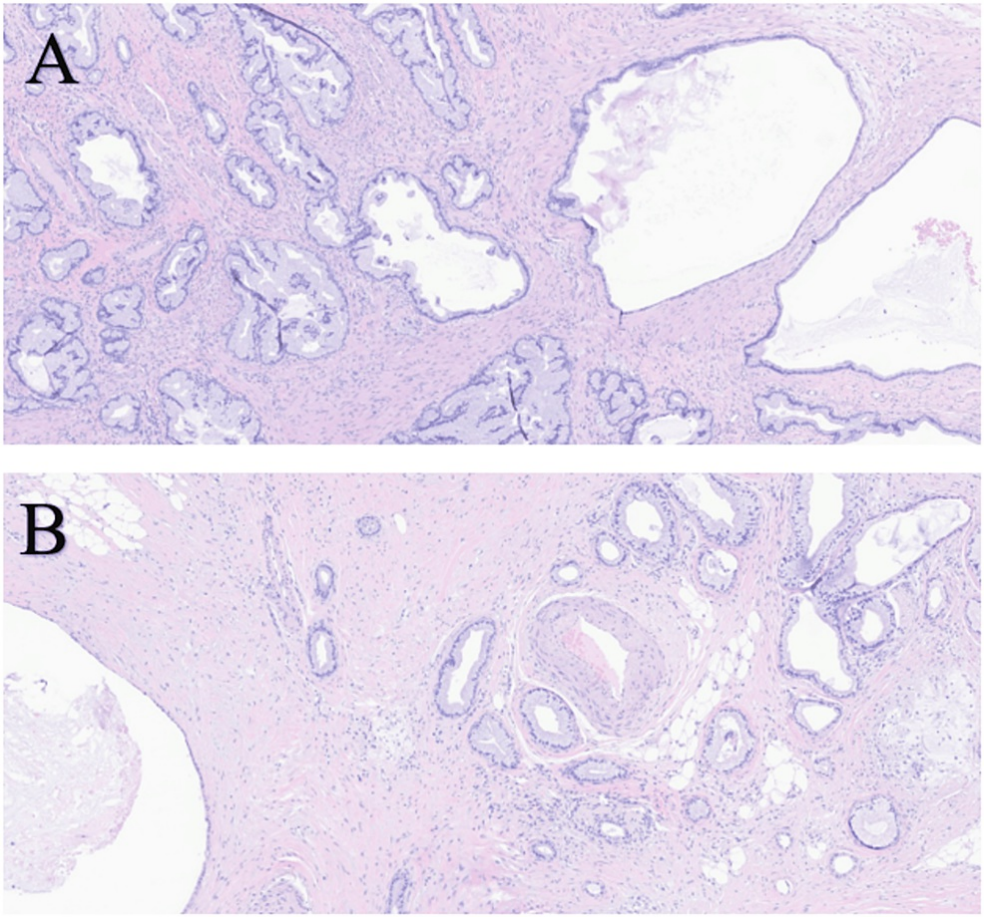
Metastatic adenocarcinoma of the pancreas. A) Dilated malignant glands and cystic lesions involving the muscularis propria and subserosa tissue of the stomach. B) Malignant glands and mucinous cyst involving the muscularis propria and subserosal adipose tissue of the small bowel. H&E staining, magnification: A) 5/40× and B) 6.3/40×. H&E: hematoxylin and eosin

Circulating tumor DNA (ctDNA) in blood was ordered to look for targetable mutations but did not reveal any somatic alterations, microsatellite instability was not detected, and tumor mutational burden (TMB) was non-evaluable. A CT-guided biopsy of LUQ abdominal nodule performed for molecular testing was negative for V-Raf murine sarcoma viral oncogene homolog B (BRAF) V600E, tropomyosin receptor kinases (TRK) A/B/C, and programmed death-ligand 1 (PD-L1). Due to strong family history of cancer, germline genetic testing from blood was performed but came back negative. The patient continued gemcitabine/protein-bound paclitaxel, and three months later, follow-up MRI showed stable disease, and CA19-9 dropped (258 U/mL; reference limit: <35 U/mL). Given that her disease was limited to the peritoneum and that she maintained excellent performance status and was motivated for treatment, her case was discussed at the tumor board, and a decision was made to proceed with cytoreductive surgery (partial gastrectomy, small bowel resection, and omentectomy) and hyperthermic intraperitoneal chemotherapy (HIPEC) with mitomycin C 30 mg and cisplatin 171 mg for 90 minutes and outflow temperature of 41°C-42°C. Surgical pathology showed metastatic adenocarcinoma involving portions of the stomach, omentum, and distal ileum (Figure 3).

**Figure 4:**
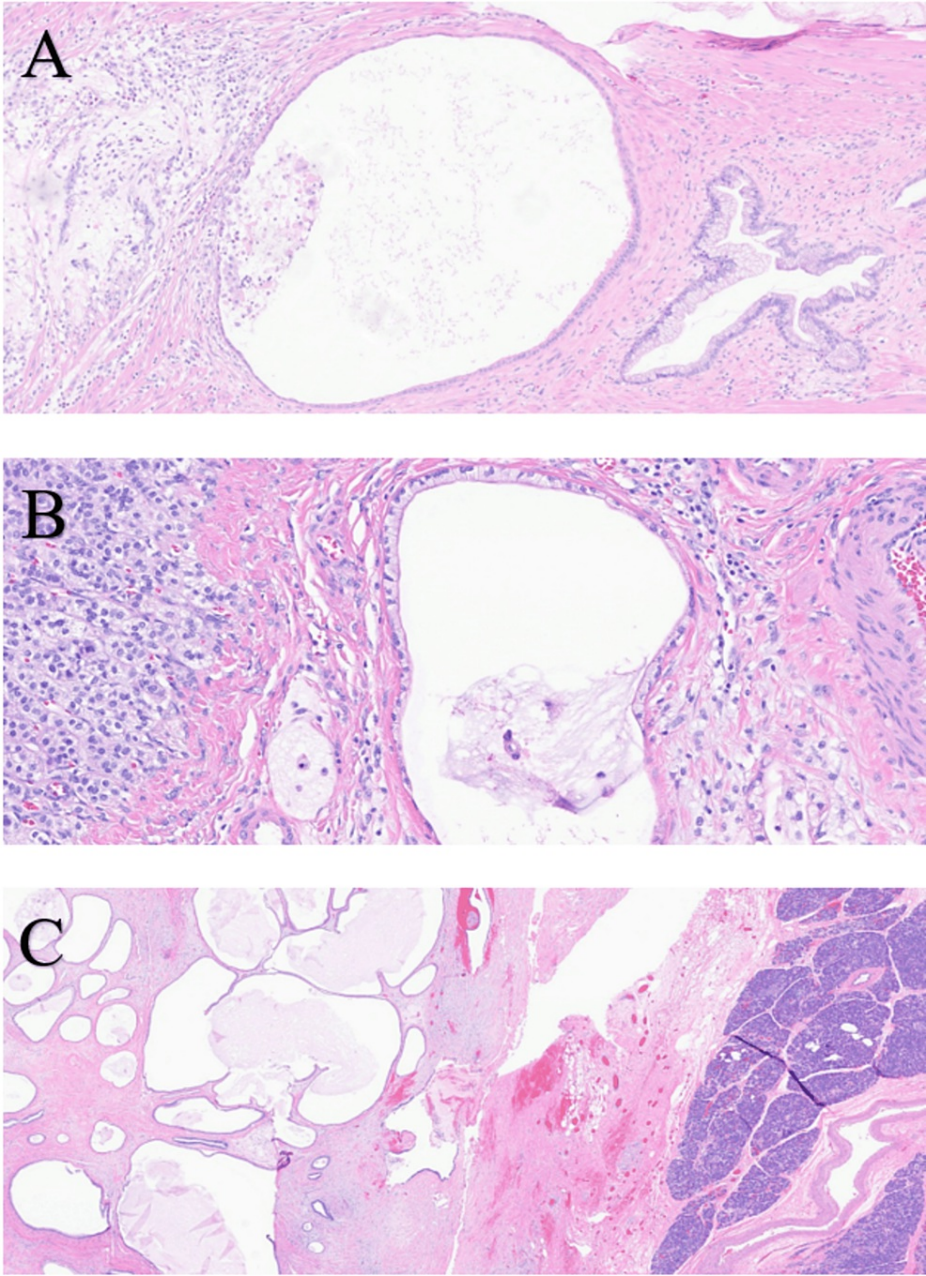
Invasive well-differentiated adenocarcinoma. A) Dilated neoplastic gland and cystic lesion lined by mucin-producing tumor cells invade the muscularis propria of the small bowel. B) Neoplastic cyst abuts the left adrenal gland. C) Multiple mucinous cysts abutting the pancreas. H&E staining, magnification: A) 10/40×, B) 20/40×, and C) 5/40×. H&E: hematoxylin and eosin

The peritoneal cancer index (PCI) score was 9. The patient recovered well and started oral maintenance chemotherapy with capecitabine 1000 mg twice per day (BID) for the remaining disease in the pancreatic bed, which could not be resected due to the risk of pancreas leak. After three months, her follow-up scans showed stable soft tissue implant in the pancreatic bed and adjacent nodule abutting the left adrenal gland, so her case was discussed again at the tumor board for a curative intervention. It was decided to stop chemotherapy and undergo surgical resection with intraoperative radiation therapy (IORT). Six months later, the patient underwent distal pancreatectomy, partial duodenectomy, partial left adrenalectomy, cholecystectomy, and IORT. Surgical pathology showed invasive well-differentiated mucinous adenocarcinoma forming a 5.3 cm ill-defined soft tissue mass adjacent to the pancreas; the tumor invades the muscularis propria of the small bowel and abuts the left adrenal gland (Figure 4).

Pancreatic and duodenal margins were negative for tumor, and a single celiac lymph node out of the six removed was positive. The patient is currently in excellent status and achieved remission.

## Discussion

PDAC is the most common histological subtype of PC [4]; it presents as a solid, ill-defined mass characterized by rapid progression and infiltration [5]. However, PDAC can have cystic changes that mimic intraductal papillary mucinous neoplasm (IPMN) on imaging. LDA is a rare variant of PDAC characterized by the microcystic ectasia of the invasive glands [6]. It is slightly more common in females and tends to involve the tail more frequently when compared to conventional PDAC [3]. On CT imaging with contrast, LDA is seen as a solid lesion with a cluster of small intratumoral cysts that are difficult to detect [7]. In contrast to branch-duct-type IPMN, LDA does not show the classic “bunch of grapes” pattern on imaging [8], but it can show the “honeycomb appearance” typical of serous cystadenoma [9]. EUS-FNA is highly sensitive in diagnosing PDAC [10] but faces challenges in differentiating LDA from IPMN, necessitating a surgical biopsy in most cases to confirm the diagnosis. Microscopically, LDA is characterized by irregularly distributed dilated glands lined by mucinous columnar cells that have irregular, basally located nuclei and foamy, microvesicular cytoplasm [11]. Immunohistochemistry (IHC) can be a useful tool in differentiating LDA from other cystic lesions. Mucin short variant S1 (MUC1), mucin 5AC (MUC5AC), and mucin 6 (MUC6) showed high positivity rates in LDA [11,12] unlike gastric-type IPMN, which is positive for MUC5AC but negative for MUC1 [13]. Genetic analysis found higher rates of KRAS (codon 12) mutation, and IHC showed the overexpression of p53 and human epidermal growth factor receptor-2 (HER2)-neu in LDA when compared to ordinary PDAC, but this was not statistically significant [3]. A more comprehensive genetic analysis is needed for this unique subtype.

Very few cases of LDA variant have been reported in the literature. Another case reported by Hara et al. described an 82-year-old female presenting with upper abdominal pain and elevated CA19-9 (185 U/mL; reference limit: ≤35 U/mL). Contrast-enhanced CT scan showed a 5.2 × 3.7 cm ill-defined multilocular cystic lesion with cyst wall showing gradual enhancement on arterial, portal, and equilibrium phases, as well as “honeycomb appearance” on T2-weighted MRI. The patient underwent distal pancreatectomy, and pathology revealed dilated malignant glands surrounded by fibrotic stroma, supporting the diagnosis of LDA [14]. The patient went on surveillance, and 13 months later, her CT scan showed a hypoattenuating lesion in the head of the pancreas. Consequently, the patient underwent a pylorus-preserving pancreatoduodenectomy showing similar microscopic features previously seen in the distal pancreas. Three months later, imaging did not reveal any evidence of recurrence.

The prognosis of LDA is similar to that of classic PDAC but drastically worse than IPMN [15]. Therefore, the differentiation of LDA from other cystic lesions is crucial. Systemic chemotherapy is currently the only approach for advanced classic PDAC, but in LDA, we found that surgical resection supplemented with chemotherapy and radiotherapy provided the best patient outcome and helped the patient achieve remission.

## Conclusions

We believe that there needs to be a new paradigm shift in the diagnosis and management of patients with LDA, focusing on a multidisciplinary approach in lieu of our current standard of care approach with only chemotherapy for advanced disease.
